# Transitioning to active-controlled trials to evaluate cardiovascular safety and efficacy of medications for type 2 diabetes

**DOI:** 10.1186/s12933-022-01601-w

**Published:** 2022-08-24

**Authors:** Darren K. McGuire, David D’Alessio, Stephen J. Nicholls, Steven E. Nissen, Jeffrey S. Riesmeyer, Imre Pavo, Shanthi Sethuraman, Cory R. Heilmann, John J. Kaiser, Govinda J. Weerakkody

**Affiliations:** 1 Division of Cardiology, University of Texas Southwestern Medical Center and Parkland Health and Hospital System, 5323 Harry Hines Blvd, Dallas, TX 75235-8830 USA; 2 Division of Endocrinology, Duke University Medical Center, Durham, NC USA; 3 Monash Cardiovascular Research Centre, Victorian Heart Institute, Monash University, Melbourne, Australia; 4 Cleveland Clinic Coordinating Center for Clinical Research, Cleveland Clinic, Cleveland, OH USA; 5 Eli Lilly and Company, Indianapolis, IN USA; 6 Eli Lilly Regional Operations GmbH, Vienna, Austria

**Keywords:** Type 2 diabetes mellitus, Antihyperglycemic, Cardiovascular, Non-inferiority trial, Imputed placebo

## Abstract

Cardiovascular (CV) outcome trials (CVOTs) of type 2 diabetes mellitus (T2DM) therapies have mostly used randomized comparison with placebo to demonstrate non-inferiority to establish that the investigational drug does not increase CV risk. Recently, several glucagon-like peptide 1 receptor agonists (GLP-1 RA) and sodium glucose cotransporter 2 inhibitors (SGLT-2i) demonstrated reduced CV risk. Consequently, future T2DM therapy trials could face new ethical and clinical challenges if CVOTs continue with the traditional, placebo-controlled design. To address this challenge, here we review the methodologic considerations in transitioning to active-controlled CVOTs and describe the statistical design of a CVOT to assess non-inferiority versus an active comparator and if non-inferiority is proven, using novel methods to assess for superiority versus an imputed placebo. Specifically, as an example of such methodology, we introduce the statistical considerations used for the design of the “Effect of Tirzepatide versus Dulaglutide on Major Adverse Cardiovascular Events (MACE) in Patients with Type 2 Diabetes” trial (SURPASS CVOT). It is the first active-controlled CVOT assessing antihyperglycemic therapy in patients with T2DM designed to demonstrate CV efficacy of the investigational drug, tirzepatide, a dual glucose-dependent insulinotropic polypeptide and GLP-1 RA, by establishing non-inferiority to an active comparator with proven CV efficacy, dulaglutide. To determine the efficacy margin for the hazard ratio, tirzepatide versus dulaglutide, for the composite CV outcome of death, myocardial infarction, or stroke (MACE-3), which is required to claim superiority versus an imputed placebo, the lower bound of efficacy of dulaglutide compared with placebo was estimated using a hierarchical Bayesian meta-analysis of placebo-controlled CVOTs of GLP-1 RAs. SURPASS CVOT was designed so that when the observed upper bound of the 95% confidence interval of the hazard ratio is less than the lower bound of efficacy of dulaglutide, it demonstrates non-inferiority to dulaglutide by preserving at least 50% of the CV benefit of dulaglutide as well as statistical superiority of tirzepatide to a theoretical placebo (imputed placebo analysis). The presented methods adding imputed placebo comparison for efficacy assessment may serve as a model for the statistical design of future active-controlled CVOTs.

**Supplementary Information:**

The online version contains supplementary material available at 10.1186/s12933-022-01601-w.

## Introduction

In 2008, the U.S. Food and Drug Administration (FDA) and the European Medicines Agency (EMA) issued guidance for the evaluation of cardiovascular (CV) safety of new antihyperglycemic therapies for type 2 diabetes mellitus (T2DM) [1, 2]. Based on this guidance, new investigational drugs for the treatment of T2DM were required to demonstrate no increased risk of major adverse CV events (MACE) compared with placebo. The standard for CV safety was statistically defined by achieving a relative risk compared with placebo, with an upper bound of 95% confidence interval (CI) of less than 1.8 for initial application and 1.3 for final approval.

Over the past decade, results from CV outcome trials (CVOTs) evaluating several new medication classes for T2DM such as dipeptidyl peptidase-4 inhibitors (DPP-4i), basal insulins, alpha-glucosidase inhibitors, glucagon-like peptide-1 receptor agonists (GLP-1 RA), and sodium glucose cotransporter-2 inhibitors (SGLT-2i) have been reported [3–9]. Only two of these trials [8, 9] compared the investigational medications with antihyperglycemic medications as active comparators, which were added to standard clinical care of patients with T2DM and established atherosclerotic cardiovascular disease (ASCVD) or multiple CV risk factors. In addition, a pragmatic open-label randomized active comparator TOSCA.IT trial evaluated CV outcomes of pioglitazone compared with sulfonylureas (glibenclamide, glimepiride or gliclazide) in patients with T2DM [10]. None of these active comparators (glimepiride, glibenclamide, gliclazide nor insulin glargine) had proven cardiovascular efficacy before their use in these CVOTs. However, numerous trials of SGLT-2i and GLP-1 RA medications have demonstrated reduction in MACE and/or fewer hospitalizations due to heart failure and/or reduction in risk for kidney disease progression [3–7, 11–14]. These results satisfied the regulatory requirements for evidence of CV safety and furthermore, their superior CV and kidney efficacy provided the basis for indications to use these agents to reduce CV and/or kidney disease risk.

The demonstration of beneficial effects of antihyperglycemic medications on CV risk in patients with T2DM has been widely recognized as a major advance, changing the clinical practice guidelines and consensus, recommendations of professional societies to emphasize prioritized use of SGLT-2i and GLP-1 RA in patients with or at high risk of CV disease [15–19]. This favorable new therapeutic landscape, on the other hand, presents significant challenges for the design, conduct, and interpretation of future CVOTs for antihyperglycemic medications. Key among these considerations include the use of a placebo control when antihyperglycemic medications with proven CV benefits are now available. For example, patients entering a placebo-controlled randomized trial who are randomized to placebo will not be allowed to receive GLP-1 RA during the trial, which may not be considered ethical nowadays. Moreover, the open-label use of other cardioprotective antihyperglycemic medications that mitigate CV risk in either trial arm as part of standard medical care could influence the primary outcome of the CVOT assessment. On the other hand, if members of new drug classes with unknown effects on CV outcomes are tested, placebo-controlled design (added to the best standard of care) may remain an important strategy to establish CV safety and efficacy. As T2DM CVOTs aim for similarly effective glycemic control in investigational and comparator groups, it is more likely that additional antihyperglycemic agents with CV efficacy will be added to patients receiving placebo. If the added medications are SGLT-2i and/or GLP-1 RA, both of which would be expected to decrease MACE, the intention-to-treat analysis of the safety and efficacy of the investigational medication would be confounded. Thus, the results may underestimate the potential benefit of the investigational therapy and may adversely influence interpretation of the safety and efficacy assessments.

An alternative to a placebo-controlled CVOT to demonstrate CV safety and/or benefit of a new diabetes medication could be direct randomized comparison of the investigational therapy with a medication that has established CV efficacy. As one of many examples, a similar approach has been used in trials to test new factor Xa inhibitor anticoagulant therapies versus the established anticoagulant therapy warfarin for stroke prevention [20–23]. Using an active comparator addresses the clinical and ethical concerns of a placebo-controlled trial as it provides high-risk participants in the control group with a proven treatment. The efficacy of anticoagulant therapies (with approximately 50%-70% reduction in risk of stroke) is however larger than the reduction in MACE risk by diabetes drugs with proven CV efficacy, which further complicates the statistical design of active-controlled diabetes CVOTs.

The evaluation of a superior treatment effect between the investigational versus diabetes drugs with proven CV efficacy, considering the relatively small but clinically meaningful risk reduction, would require larger sample size and/or longer trial duration to ascertain large number of events for adequate statistical power. For example, a diabetes CVOT designed with 90% power to demonstrate 10% greater relative risk reduction for an investigational therapy over an active comparator would require an accrual of 3786 primary outcomes. Assuming an annualized MACE event rate of 4%, this would approximate to 100,000 patient-years of exposure (e.g., 25,000 patients followed for an average of four years). A successful outcome in this setting would prove superiority of the investigational medication compared with active control, and by extension also in relation to placebo.

An alternative objective of an active-controlled diabetes CVOT could be to demonstrate superiority to an imputed placebo by demonstrating NI to a proven active comparator using sufficiently narrow NI margins. This approach has the advantage of a smaller sample size and/or shorter duration of the trial. Assuming that the comparator is proven to reduce CV relative risk by at least 5%, a NI margin of 1.05 could be justified. Using the same assumption for an annualized MACE event rate of 4% as above and accepting the NI margin of up to 1.05 to establish NI to the active comparator, the study with 1600 MACE events, less than half the size as the trial outlined above with direct superiority comparison with the active comparator would also establish CV efficacy versus an imputed placebo.

One example for the indirect demonstration of superiority versus imputed placebo is the International Joint Efficacy Comparison of Thrombolytics (INJECT) randomized, double-blind CVOT [24] that compared reteplase double-bolus administration with streptokinase in acute myocardial infarction (MI) and was designed to show imputed CV efficacy by demonstrating NI of reteplase to streptokinase for acute MI. While the trials comparing direct oral anticoagulants to warfarin for the treatment of atrial fibrillation did not use this method, the methodology has been applied posthoc across the family of trials to illustrate the methodology [25]. Such imputed placebo comparisons to date, to our knowledge, have not been used to support regulatory product labeling for indication of a medication as is the case with the SURPASS trial.

There is no precedent so far for the evaluation of an antihyperglycemic medication in T2DM for a CV risk reduction indication based on a CVOT designed to test NI versus an active comparator with proven CV efficacy due to the relatively recent demonstration of CV and kidney efficacy of SGLT-2is and GLP-1 RAs. In addition, the modest estimates of relative risk reduction of MACE by SGLT-2is and GLP-1 RAs also complicate such designs given the narrow NI margins required to underpin imputed placebo comparison.

Tirzepatide is a novel, once-weekly, injectable, dual glucose-dependent insulinotropic polypeptide (GIP) and GLP-1 RA, is being developed for the treatment of type 2 diabetes (T2D) and obesity [26] with CV safety demonstrated in the late phase clinical program [27, 28]. The ‘Effect of Tirzepatide versus Dulaglutide on Major Adverse Cardiovascular Events (MACE) in Patients with Type 2 Diabetes (SURPASS CVOT)’ trial is the first trial designed to evaluate the CV efficacy of a novel antihyperglycemic medication for T2DM compared with a blinded active comparator with proven, product labeled CV efficacy, dulaglutide. The development of the SURPASS CVOT statistical analysis plan is summarized here to review the methodology and analytic considerations of a trial designed to prove NI versus active comparator, with the novel addition of using imputed placebo method to assess superiority.

### Defining the NI margin for a CVOT with an active comparator

For an active-controlled trial developed with a view of establishing CV efficacy, the determination of the NI margin is critical [29], and must be established a priori to ensure that the investigational therapy (a) would be superior to placebo if a placebo group had been included in the trial (referred to as margin 1, or “M1” in FDA guidance); and (b) is non-inferior to the active comparator (referred to as margin 2, or “M2” in FDA guidance). This latter, more stringent requirement ensures that trial success is declared only if an investigational therapy demonstrates both NI to the active comparator as well as superiority to an imputed placebo in integrated data analyses.

#### Establishing superiority of an investigational therapy to a putative placebo

Establishing whether an investigational therapy is superior to a putative placebo (i.e., hazard ratio (HR) [investigational therapy versus putative placebo] < 1.0), in an active comparator trial uses estimates of efficacy from (a) direct comparison of the efficacy of the investigational therapy and active comparator in the trial (HR of investigational therapy versus active comparator), and (b) comparison of the efficacy of the active comparator and placebo imputed from prior trials (HR of the active comparator versus placebo). One approach to estimate the HR (investigational therapy versus putative placebo) is simply multiplying the hazard ratios: HR (investigational therapy versus active comparator) × HR (active comparator versus placebo). Therefore, demonstrating that the HR of the investigational therapy versus an active comparator is less than the inverse of HR of the active comparator versus placebo with high probability ensures superiority to a putative placebo. This approach has been used previously in posthoc analyses of direct oral anticoagulants (DOAC) versus warfarin trials to assess the superiority of DOACs versus imputed placebo [25], and related statistical methods and limitations have been described, but to our knowledge not previously used to support regulatory medicinal product labeling considerations [30–32].

To account for variability in the efficacy of the active comparator in any new trial compared with previous placebo-controlled trials, FDA guidance recommends a more conservative approach for establishing superiority of the investigational therapy to a putative placebo. The standard approach to setting the M1 NI margin is to use the inverse of a conservative estimate of active comparator versus placebo, typically the upper bound of the 95% CI of efficacy of the active comparator (HR of active comparator versus placebo) estimated from pooled/meta-analyses of previous trials [29]. Stated differently, if the upper bound of the 95% CI for the HR (investigational therapy versus active comparator) is less than the inverse of upper bound of the 95% CI for the HR (active comparator versus placebo), the investigational therapy would be interpreted as superior relative to a putative placebo. When more than one placebo-controlled trial of the active comparator exists or several placebo-controlled trials of medications from the same pharmacological class have been conducted, a conservative estimate of the HR (active comparator versus placebo) can be derived using a meta-analysis of studies with similar design features [30, 33]. Study design features for consideration may include patient population, trial duration, event rates, and comparable inclusion or exclusion criteria. For example, the Stroke Prevention Using Oral Thrombin Inhibitor in Atrial Fibrillation V (SPORTIF V) CVOT evaluated NI of ximelagatran compared with warfarin on the occurrence of stroke in patients with non-valvular atrial fibrillation [34]. In this case, the NI margin was established by a meta-analysis of six trials comparing warfarin with placebo for stroke or embolic event risk. This analysis showed a benefit of warfarin versus placebo with a HR of 0.36 and 95% CI of 0.25–0.53 [29]. Therefore, in the SPORTIF V CVOT [34], an upper bound for the NI margin was set as 1.9 (1/0.53).

#### Demonstrating non-inferiority of investigational therapy to the active comparator (Determination of the non-inferiority margin – M2)

The value of the M1 NI margin is set to ensure that the investigational therapy is superior to a putative placebo. However, if the M1 NI margin is too large, an investigational therapy may meet the mark for superiority to placebo without showing convincing NI to the active comparator. For example, due to the overwhelming efficacy of warfarin versus placebo, in the SPORTIF V CVOT [34], the M1 NI margin of 1.9 (i.e., the inverse of the efficacy of warfarin) sets a very wide margin for comparing ximelagatran and warfarin. If the M1 level was used alone, NI could be claimed for ximelagatran versus warfarin with an observed HR substantially > 1.0, raising the possibility that it had inferior efficacy for stroke risk compared with warfarin. To minimize the possibility of such an outcome, it is usually required that the investigational therapy preserves a minimal clinically acceptable difference in efficacy to the active comparator. The M2 NI margin or the minimal clinically acceptable difference in efficacy between the investigational therapy and active comparator relies in part on clinical judgment. FDA guidance suggests that determination of the M2 NI margin be guided by preservation of a pre-specified fraction of the efficacy of the active comparator compared to placebo. In general, it is assumed that preservation of at least 50% of the efficacy of the active comparator is appropriate to claim NI in CVOTs [29]. Accordingly, in the series of NI trials comparing the various DOACs with warfarin, M2 was chosen to be the square root of M1 [25], and for example in SPORTIF V CVOT [34], the M2 was chosen to be square root of 1.9, i.e., 1.378. This was to ensure ximelagatran preserves at least 50% of the efficacy of warfarin, the upper bound of 95% CI of ximelagatran versus warfarin needed to be less than 1.378 to demonstrate NI. In the SPORTIF V study, ximelagatran did not establish NI to warfarin since the upper bound of two-sided 95% CI was 2.21, exceeding the required threshold of 1.378 [29]. These methods have become standard and have been used routinely for registration trials of drugs to support claims of NI versus proven active comparators.

Regulatory guidance for NI [29] is generally presented in a frequentist point of view, where efficacy is viewed as a fixed value. Efficacy of the active comparator versus placebo may be viewed as a random variable as opposed to a fixed value. In this setting, the conservative estimate of the HR of active control versus placebo obtained from a Bayesian hierarchical meta-analysis of placebo-controlled trials of active control or from placebo-controlled trials from medications for the same class may be chosen as M1. Furthermore, a Bayesian approach can be used for combining distribution of efficacy of active control versus placebo together with the data from active controlled trial to assess the fraction of efficacy of the active control preserved by the investigational agent.

### Statistical design of an active comparator CVOT to determine MACE efficacy for a new antihyperglycemic medication in patients with T2DM

To adapt to the current landscape of T2DM therapy, the SURPASS CVOT was designed to establish NI of tirzepatide to dulaglutide (a GLP-1 RA with proven CV efficacy), with the novel addition of a planned imputed placebo comparison to evaluate for superiority of CV efficacy. Dulaglutide was found superior to placebo in reducing MACE in The Researching Cardiovascular Events With a Weekly INcretin in Diabetes (REWIND) trial [3], a CVOT in patients with T2DM with or without established ASCVD; this result is now reflected in a product labeled indication. The REWIND trial evaluated CV outcomes of dulaglutide versus placebo, enrolling 9901 participants with or at high risk for atherosclerotic CV disease (mean age of 66.2 years, and a median follow-up of 5.4 years), demonstrating superiority of dulaglutide (HR 0.88; 95% CI 0.79–0.99) for the primary composite outcome of CV death, MI, or stroke. A subgroup analysis showed that the presence or absence of prevalent ASCVD did not modify the treatment efficacy of dulaglutide [3]. To account for possible variability in the trial-to-trial efficacy of dulaglutide, and variability in the medication-to-medication efficacy across the GLP-1 RA class, the distribution of CV risk reduction was generated from results of completed placebo-controlled GLP-1 RA trials (Table 1). As the SURPASS CVOT is conducted in patients with established ASCVD, the patient subgroups with prevalent ASCVD from REWIND and other comparable GLP-1 RA trials were used.

**Table 1:** Summary of completed GLP-1 RA cardiovascular outcome trials for the meta-analysis – primary outcomes and associated hazard ratio in patients with a history of atherosclerotic cardiovascular disease (ASCVD)

Study	Medication	Treatment	Number of patients with MACE-3	Hazard ratio (95% CI)
EXSCEL [36]	Exenatide	Active	722	0.90 (0.82, 1.00)
		Placebo	786	
		Total	1508	
LEADER [6]	Liraglutide	Active	536	0.83 (0.74, 0.93)
		Placebo	629	
		Total	1165	
HARMONY [37]	Albiglutide	Active	338	0.78 (0.68, 0.90)
		Placebo	428	
		Total	766	
SUSTAIN-6 [5]	Semaglutide	Active	98	0.72 (0.55, 0.93)
		Placebo	137	
		Total	235	
PIONEER-6 [4]	Semaglutide	Active	57	0.83 (0.58, 1.17)
		Placebo	68	
		Total	125	
REWIND [3]	Dulaglutide	Active	280	0.87 (0.74,1.02)
		Placebo	315	
		Total	595	

*CI* confidence interval, *EXSCEL* Exenatide Study of Cardiovascular Event Lowering, *GLP-1 RA* glucagon-like peptide-1 receptor agonist, *LEADER* Liraglutide Effect and Action in Diabetes: Evaluation of cardiovascular outcome Results, *MACE* major adverse cardiovascular events (a composite outcome comprising non-fatal myocardial infarction, non-fatal stroke, and death from cardiovascular causes), *PIONEER* Pre-operative wIndOw study of letrozole plus PR agonist (Megestrol Acetate) versus letrozole aloNE in post-menopausal patients with ER-positive breast cancer, *REWIND* Researching Cardiovascular Events With a Weekly Incretin in Diabetes, *SUSTAIN* Semaglutide Unabated Sustainability in Treatment of Type 2 Diabetes

**Fig. 1:**
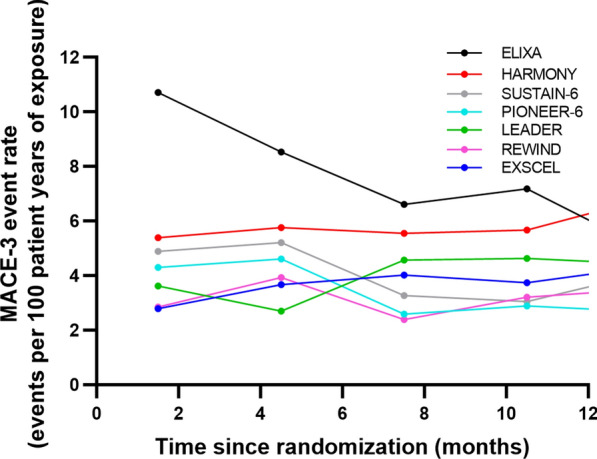
MACE outcome event rate over time in different cardiovascular outcome trials. *ELIXA* Evaluation of Lixisenatide in Acute Coronary Syndrome, *EXSCEL* Exenatide Study of Cardiovascular Event Lowering, *GLP-1 RA* glucagon-like peptide-1 receptor agonist, *LEADER* Liraglutide Effect and Action in Diabetes: Evaluation of cardiovascular outcome Results, *MACE* major adverse cardiovascular events (a composite outcome comprising non-fatal myocardial infarction, non-fatal stroke, and death from cardiovascular causes), *PIONEER* Pre-operative wIndOw study of letrozole plus PR agonist (Megestrol Acetate) versus letrozole aloNE in post-menopausal patients with ER-positive breast cancer, *REWIND* Researching Cardiovascular Events With a Weekly Incretin in Diabetes, *SUSTAIN* Semaglutide Unabated Sustainability in Treatment of Type 2 Diabetes

In accordance with FDA guidance [27], for SURPASS CVOT, the NI margin was determined from an estimate of the HR (GLP-1 RA versus placebo) relative to MACE risk using a Bayesian hierarchical meta-analytic model (see supplemental information) for patients with established ASCVD. This analysis combined aggregate data from all available CVOTs described in Table 1. Exclusion of the ELIXA CVOT [36] was justified based on differences in the patient population from all other included trials and pharmacologic characteristics of lixisenatide compared with other GLP-1 RAs (see Fig. 1), as has also been done in published meta-analyses [37].

The meta-analysis established the 50th percentile (median) of 0.85 and 97.5th percentile of 0.95 ensuring that the HR of dulaglutide versus placebo is less than 0.95 with a probability of 0.975. Accordingly, 1/0.95 = 1.05 was used as the pre-specified M1 NI margin in the SURPASS CVOT statistical analysis plan to demonstrate superiority of tirzepatide relative to a putative placebo.

The SURPASS CVOT trial was designed to accrue 1600 primary outcome events comprising the time to the first event of CV death, MI or stroke and provides approximately 90% power to establish superiority to a putative placebo with an assumed 10% lower MACE risk for tirzepatide compared with dulaglutide and a NI boundary of 1.05.

**Fig. 2:**
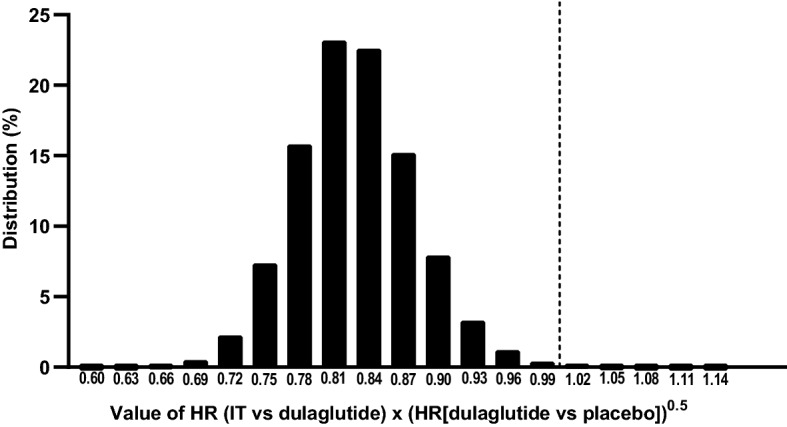
Demonstrating Preservation of 50% Efficacy of Dulaglutide. *HR* hazard ratio, *IT* investigational treatment. The simulation study showed a probability of 0.997 for tirzepatide to preserve 50% of dulaglutide efficacy. Therefore, due to the conservative choice of M1 margin and large sample size of SURPASS CVOT, a NI margin of 1.05 in the SURPASS CVOT will not only demonstrate NI of tirzepatide to dulaglutide, but also superiority of tirzepatide to a putative placebo

Figure 2 illustrates the distribution of the HR (tirzepatide versus dulaglutide) × HR (dulaglutide versus placebo)^(0.5)^ evaluated in a simulation study. In this simulation study, conducted to assess the probability that tirzepatide preserving 50% of the efficacy of dulaglutide, the distribution of efficacy of dulaglutide versus placebo is represented by the distribution of HR (GLP-1 RA versus placebo) obtained using the Bayesian hierarchical meta-analytic model described previously and the distribution of HR of tirzepatide versus dulaglutide is developed under the assumption of an observed HR of tirzepatide versus dulaglutide of 0.95, the maximum value needed to meet the requirement for tirzepatide to be superior to a putative placebo in SURPASS CVOT (see Supplemental Information).

## Discussion

Here the statistical assumptions and analytic methodology have been described that underlie the analytic approach used to design an active comparator trial, the SURPASS CVOT, designed not only to assess NI of tirzepatide versus the proven active comparator dulaglutide, but if NI is demonstrated, to also assess superiority of tirzepatide versus placebo, capitalizing on integrated data analyses to prove efficacy versus an imputed placebo. This methodology could serve as a model for future active-controlled CVOTs evaluating safety and efficacy of new antihyperglycemic medications for T2DM.

Demonstrating CV efficacy versus placebo for an investigational therapy in an active-controlled trial of a proven therapy minimally requires pre-specification of a NI margin based on integrated data analyses of existing data for the active comparator versus placebo to statistically demonstrate superiority against a putative placebo. In the statistical design of the SURPASS CVOT, such a threshold has been derived from a meta-analysis of placebo-controlled GLP-1 RA CVOTs. Bayesian methods were used to determine a NI margin that would establish NI of tirzepatide to dulaglutide and would also be adequate to establish the superiority of tirzepatide versus a putative placebo.

After reviewing the results of CVOTs of antihyperglycemic medications for T2DM in the 12 years since issuing their 2008 guidance, the FDA recently issued a new draft guidance removing pre- and post-marketing requirements for medications narrowly focused on excluding increased CV risk [30]. However, with the demonstration that some antihyperglycemic medications have CV benefits, evaluating therapies in outcomes trials of T2DM remains an important area of clinical investigation with major considerations with respect to the ethics of conducting placebo-controlled trials. It is now ethically indefensible to restrict background use of antihyperglycemic therapies that are proven to reduce CV for eligible patients, as these medications are now recommended as standard-of-care. Thus, present and future CVOTs of T2DM medications will need to compare investigational therapies directly with agents that have known benefits on the incidence of MACE, heart failure, and/or kidney disease progression.

Few limitations apply to the current study. The meta-analysis was conducted using published aggregate data. Meta-analyses of individual patient data are always preferred over trial summary level analyses and may improve precision of estimates and better account for differences in net exposure and duration of follow-up. However, ascertainment of patient-level data across trials is extremely complex and often not possible; and within the GLP-1 RA arena, summary level analyses have largely been consistent with individual trial results [37]. Additionally, an important assumption is that the efficacy of dulaglutide versus placebo demonstrated in the REWIND trial remains unchanged despite changing background care. The ‘best standard of care’ is evolving with time as numerous cardioprotective drug classes have been introduced to clinical practice in the last decade. The interaction of these drugs used as background care with any GLP-1 RA including dulaglutide on CV outcomes is not fully evaluated. Due to the different mechanism of actions (MOAs) of these drug classes when compared with GLP-1 RA, a meaningful interaction with the efficacy of GLP-1 RAs, including dulaglutide, is unlikely.

In summary, the rapidly evolving therapeutic environment for T2DM has provided new options for patients that can further mitigate risk for CV disease complications, which remains a major cause of mortality and morbidity in this population. However, these developments require new considerations for present and future CVOTs that will invariably necessitate the use active comparators with proven clinical efficacy to evaluate CV safety and incremental efficacy of novel agents. This approach requires careful consultation with regulatory agencies, but active comparator designs will become more refined and accepted as their utility increases.

## Supplementary Information

**Additional file 1. **Bayesian hierarchical meta-analytic model. Posterior distribution of HR (tirzepatide versus dulaglutide) assuming SURPASS CVOT resulted in an observed HR (tirzepatide versus dulaglutide) of 0.95.

## Data Availability

Data sharing is not applicable to this article as no datasets were generated or analyzed for the purpose of this publication.
