# Supplementary material for: Transitioning to active-controlled trials to evaluate cardiovascular safety and efficacy of medications for type 2 diabetes
Source: Cardiovasc Diabetol. 2022 Aug 24;21:163. doi: 10.1186/s12933-022-01601-w (PMC9400320; doi:10.1186/s12933-022-01601-w)
Supplement: Supplementary file 1 — Additional file 1. Bayesian hierarchical meta-analytic model. Posterior distribution of HR (tirzepatide versus dulaglutide) assuming SURPASS CVOT resulted in an observed HR (tirzepatide versus dulaglutide) of 0.95. [file 12933_2022_1601_MOESM1_ESM.docx]

**Additional file 1**

**Bayesian hierarchical meta-analytic model**

In the Bayesian meta-analytic model, it is assumed that the GLP-1 RA class reduces MACE log-transformed risk, and that each specific GLP-1 RA has a different MACE risk reduction (θ_i_) that is normally distributed around the mean GLP-1 RA effect (θ_c_).

$$y_{i}\sim N\left( \theta_{i},\sigma_{i}^{2} \right)$$

$$\theta_{i}\sim N\left( \theta_{c},\tau^{2} \right)$$

$$\theta_{c}\sim N\left( 0,4^{2} \right)$$

$$\tau^{2}\sim\Gamma^{-1}\left( 0.001,0.001 \right)$$

where ‘$y_{i}$’ is the observed log hazard ratio of study ‘i' and ‘σ_i_^2’^ is the standard error of $y_{i}$, and Γ^−1^ represents the inverse gamma distribution. Four independent Markov Chain Monte Carlo chains were used for sampling with a burn-in of 1,000 samples on each chain. A total of 400,000 samples were saved and used to back-transform the upper credible limit of the hazard ratio (GLP-1 RA relative to placebo) to the M1 non-inferiority margin limit.

**Posterior distribution of HR (tirzepatide versus dulaglutide) assuming SURPASS CVOT resulted in an observed HR (tirzepatide versus dulaglutide) of 0.95**

Suppose $\mu=log(HR\left[ tirzepatide versus dulaglutide \right])$ and prior distribution of $\mu\sim N(\mu_{0},\sigma^{2})$.

Let $\log\left( \mathrm{hr} \right)$ be the observed $log(HR\left[ tirzepatide versus dulaglutide \right])$ from SURPASS CVOT with 1600 primary outcomes. Then, the conditional distribution of $\log\left( \mathrm{hr} \right) given \mu\sim N \left( \mu, \frac{4}{1600} \right)$ and the posterior distribution of $\mu given log(hr) \sim N(\mu_{1},\sigma_{1}^{2})$, Where $\sigma_{1}^{-2}=\sigma^{-2}+\left( \frac{4}{1600} \right)^{-1}$ and $\mu_{1}= \frac{\sigma_{1}^{2}}{\sigma^{2}}\mu_{0}+ \frac{\sigma_{1}^{2}}{\left( \frac{4}{1600} \right)}log(hr).$

With 1600 primary endpoint outcomes, it can be shown that an observed HR of tirzepatide versus dulaglutide ≤0.95 in SURPASS CVOT will result in the upper bound of the 95% of the CI for the HR <1.05; meeting the requirement for tirzepatide to be superior to putative placebo. Assuming a prior with $\mu_{0}=0$ and $\sigma^{2}\to\infty, \sigma_{1}^{2}=\left( \frac{4}{1600} \right)$ and $\mu_{1}=Log(0.95).$ Thus $\mu:log(hr)\sim N \left( log(0.95), \frac{4}{1600} \right)$. Thus, an observed HR (tirzepatide versus dulaglutide) of ≤0.95 in SURPASS CVOT will result in the upper bound of the 95% CI for HR (tirzepatide versus dulaglutide) <1.05.
